# Physicochemical Evaluation of Edible Cyanobacterium *Arthrospira platensis* Collected from the South Atlantic Coast of Morocco: A Promising Source of Dietary Supplements

**DOI:** 10.1155/2021/3337231

**Published:** 2021-07-09

**Authors:** Hanane Ennaji, Mohammed Bourhia, Ikram Taouam, Aziz Falaq, Touria Ould Bellahcen, Ahmad Mohammad Salamatullah, Abdulhakeem Alzahrani, Heba Khalil Alyahya, Riaz Ullah, Samir Ibenmoussa, Naima Khlil, Mounia Cherki

**Affiliations:** ^1^Laboratory of Chemistry, Biochemistry, Nutrition, and Environment, Faculty of Medicine and Pharmacy, University Hassan II, 20000 Casablanca, Morocco; ^2^Health and Environment Laboratory, Faculty of Sciences Ain Chock, Hassan II University of Casablanca, B.P 5366 Maarif, Casablanca, Morocco; ^3^Official Laboratory for Chemical Analysis and Research, B.P 20110, Casablanca, Morocco; ^4^Department of Food Science & Nutrition, College of Food and Agricultural Sciences, King Saud University, P.O. Box 2460, Riyadh 11451, Saudi Arabia; ^5^Department of Pharmacognosy (MAPPRC), College of Pharmacy, King Saud University, P.O. Box 2457, Riyadh 11451, Saudi Arabia

## Abstract

The cyanobacterium *Arthrospira platensis (A. platensis)*—a genus of nonheterocystous filamentous cyanobacteria—is used in industrial applications and as a food supply. The current research work aims to study the physicochemical characteristics of *A. platensis* indigenous to the Moroccan Atlantic coast at Laayoune (Foum El Oued lagoon). The contents of proteins, carbohydrates, vitamins, lipids, minerals, heavy metals, energy value, humidity, ash, pigments, and tannins in *A. platensis* were investigated using protocols as described in the earlier literature. The values of protein, carbohydrate, and lipid contents in *A. platensis* were 58.9 ± 0.07, 14.67, and 45.54% respectively. The values of vitamins B_2_ and B_3_ dosed in *A. platensis* were 1.31 ± 0.19 and 30.8 ± 0.001 mg/kg, respectively. The values of heavy metals including lead and chromium were 70 ± 4.5 and 5 ± 0.5 PPB (parts-per-billion), respectively; however, no trace concerning cadmium was detected. The values of energy value, humidity, and ash content were 346.48 ± 0.21, 11.6 ± 0.17%, and 9.1 ± 0.21% kcal/100 g, respectively. The results of pigment content showed the presence of chlorophyll b, chlorophyll a, and carotenoids of 37.506 ± 3.38, 26.066 ± 3.08, and 9.52 ± 0.22 mg/g, respectively. The results obtained revealed that *A. platensis* indigenous to the Moroccan Atlantic coast at Laayoune was found to be very rich in proteins, carbohydrates, vitamins, minerals, ash, and pigments and lower in heavy metals and saturated fats when compared with species investigated in the literature. Thus, *A. platensis* indigenous to the Moroccan Atlantic coast at Laayoune fulfills the requirements for being used as dietary supplements.

## 1. Introduction

Microalgae comprising large photosynthetic plants whose vegetative system is called “thallus.” They have variable shapes and dimensions. Some of them are microscopic, and others are macroscopic, but they share structural and genetic similarities [1]. Overall, microalgae are subdivided into several classes including 30,000 to 40,000 species. Microalgae present a large morphological and physiological diversity, which helps them create an aerobic atmosphere necessary for the development of life [2]. The majority of microalgae species are micromicroalgae, which account for more than ten million [3]. Microalgae are mainly aquatic living in fresh or marine waters, and some of them on the high mountains [4]. Microalgae are recognized for their ability to withstand high temperatures in the waters of thermal springs [4].

Nowadays, microalgae seem to be one of the best solutions for producing high-quality food supplements [5]. Microalgae were the first photosynthetic living things that have appeared on the Earth about 3 to 4 billion years ago through cyanobacteria. According to their color pigments, microalgae are usually classified into green, brown, and red. As a result, these microalgae are divided into four classes: green microalgae (chlorophytes), blue microalgae (cyanobacteria), red microalgae (rhodophytes), and brown microalgae (chromophytes) (see [2] and [6]).

In recent times, the use of photosynthetic microorganisms has progressively increased. They have been used in different fields, including food dyes, cosmetics, dietetics, and biotechnology [7]. Cyanobacteria are prokaryotes that accomplish oxygenic photosynthesis and form a wide taxonomic group within eubacteria. Cyanobacteria are morphologically subdivided into unicellular or filamentous organisms. Functionally, these microorganisms can be classified into N2-fixing and non-N2-fixing. [8]. *Arthrospira* is a genus belonging to nonheterocystous filamentous cyanobacteria that live in an alkaline environment [9]. Even though these microorganisms constitute a special taxonomic unit, many *Arthrospira* species were classified in the genus *Spirulina*, and some of them are still being named under this name [10]. Anyway, the current taxonomy asserts that the name “Spirulina” used to indicate strains used as food supplements is unsuitable and there is accordance that *Arthrospira* is a distinct genus including more than 30 different species [11].

*Arthrospira* species are rich in nutrients like essential fatty acids, minerals, vitamins, and pigments [12]. Thus, they have largely been used as food supplements, feedstock in both agriculture and aquaculture [13]. It has become an interesting source of organic material, beta-carotene, and natural food dyes [14]. In addition to their important nutritional value, *Arthrospira* species have also the requirement for being introduced to serve heath by exhibiting interesting pharmacological activities like anti-inflammatory, antioxidant, and immunomodulating ([15] and [16]). *Arthrospira platensis* (*A. platensis*) is recommended for being applied in environmental sectors for wastewater treatment (metals, nitrogen, phosphorus) [16].

*Arthrospira* species have ecologically valuable criteria such as alkali and salt tolerance. This organism can grow where several species cannot even under high salt concentrations of 1.5-fold higher than seawater as reported in earlier works [17]. These photosynthetic organisms are often live in lakes with high pH and carbonate levels [18].

It was reported that the Moroccan *A. platensis* has been used in the Mediterranean diet for many decades. In this sense, *A. platensis* requires processing into an acceptable product before it can be used. However, the physicochemical composition of species indigenous to the Moroccan Atlantic coast at Laayoune (Foum El Oued lagoon) has not yet been investigated. It is thus fitting that the present research work aimed to achieve this goal by studying the physicochemical criteria of *A. platensis* collected from this local cultivar.

## 2. Material and Methods

### 2.1. Organism

**Figure 1:**
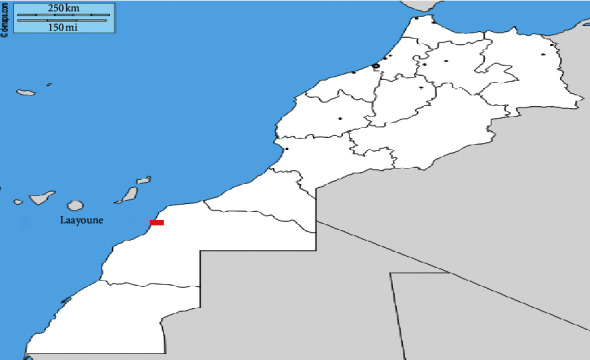
Area of collection (Laayoune-Foum El Oued lagoon-Morocco) [19].

**Figure 2:**
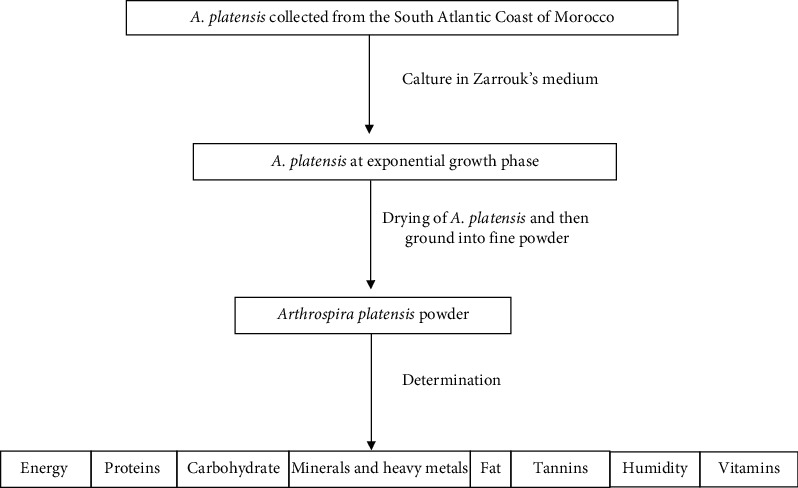
Scheme of the study design.

*Arthrospira platensis* was obtained from the culture collection at the Moroccan Foundation for Advanced Science, Innovation and Research, which was originally isolated from the Atlantic coast at Laayoune (Foum El Oued lagoon)—south of Morocco (Figure 1) (027° 06′ 00.0″ N, 013° 25′ 00.0″ W) before being cultured at the Faculty of Sciences Ain Chock, University Hassan II of Casablanca, Morocco. Briefly, the cells obtained were cultured in Zarrouk's medium (Zarrouk, 1966) at 31 ± 1°C, pH = 9, irradiated with 40 mol m−^2^ s−^1^ of cool-white fluorescent light (12-h:12-h light:dark cycle) and aerated with ambient air (360 ppmv CO_2_). Samples in the exponential growth phase were used to perform the analysis (Figure 2).

### 2.2. Physicochemical Characteristics of the Study Area (Foum El Oued Lagoon)

#### 2.2.1. Temperature

The temperature of the collection area fluctuated between 16.1°C and 17.2°C at time sampling. Lagoon water was generally warmer than that of the ocean. Similarly, the seasonal variation was pronounced, with warmer water in September (21.5–24.4°C) than in February (16.3–19.5°C) [20].

#### 2.2.2. Salinity

Salinity showed an increasing gradient from downstream to upstream. Salinity gradually increased inside the lagoon with values close to those of the ocean (34–35 PSU). Salinity was higher in September than in February as reported in earlier works [20].

#### 2.2.3. Dissolved Oxygen

Dissolved oxygen concentrations were variable according to stations (6.9–8.5 mg l^−1^). A strong concentration gradient was noted from downstream to upstream in the lagoon [20].

#### 2.2.4. Nitrates

The lagoon was found to be generally richer in nitrates in February than in September. The concentration of nitrates decreased from downstream to upstream in the lagoon with values ranging from 80 *µ*g l^−1^ (H4) to 9.9 *µ*g l^−1^ [20]_._

#### 2.2.5. Phosphates

The spatial distribution of phosphates showed two trends depending on the tide and the season. This distribution was more homogeneous across the lagoon with 97 *µ*g l^−1^, and the water was generally richer in phosphates in February than in September as reported elsewhere [20]_._

### 2.3. Protein Content Determination

#### 2.3.1. Quantitative Determination

A zero-point seventy-five gram of *A. platensis* dried sample was introduced into a flask containing 7.5 g of catalyst (100 g of potassium sulfate K_2_SO_4_, 10 g of copper sulfate CuSO_4_.5H_2_O) and 15 mL of sulfuric acid H_2_SO_4_ (0.1 N). The assay was carried out in duplicate. The mixture was subjected to mineralization using a mineralization ramp apparatus (Büchi) for 4 hours until reaching a maximum production of ammonium sulfate (NH_4_)_2_SO_4_. After cooling, the volume of the mineralized sample was mixed with 50 mL of distilled water. Afterward, 85% NaOH solution (65 mL) was added to the mineralized sample before being distilled, and then the solution was trapped in boric acid (H_3_BO_3_, 4%). Next, the ammoniacal distillate was titrated with sulfuric acid (0.1 N) to perform analysis [21].

The total protein content was calculated using the following formula:

(1) $$\begin{array}{c} \text{\% of the protein content}=\frac{\left(\text{VV}\times 0.0014\times F\right)}{\text{PE}}\times 100. \end{array}$$

Here*, F*: conversion factor (6.25), VV: volume of the sulfuric acid solution, and PE: test portion.

#### 2.3.2. Qualitative Determination of Amino Acids

The determination of the amino acid composition (valine, glutamate, arginine, threonine, methionine, and phenylalanine) of *A. platensis* was carried out using high-performance liquid chromatography (HPLC). Briefly, 5 g of *A. platensis* were dried in an oven set at 40°C for 24 h before being added to 40 mL of sulfuric acid H_2_SO_4_ (2N). After maceration for 3 hours, the extract obtained was filtered before being analyzed using HPLC (Agilent 1100) (mobile phase: 43% KH_2_PO_4_ and 57% methanol; precolumn: o-phthaldialdehyde (OPA); column: C18; volume injection: 20 *µ*L; UV detection at a wavelength of 333 nm; flow rate: 0.8 mL/min) [22].

The concentration of amino acids was calculated using the following formula:

(2) $$\begin{array}{c} \left[\text{XE}\right]=\frac{\left(\left[\text{St}\right]\times \text{AE}\right)}{\text{AS}\times \text{FD}}. \end{array}$$

Here, AE: area of the sample, AS: area of the standards, FD: dilution factor, [St]: concentration of standards, and [XE]: concentration of the sample in ppm.

### 2.4. Determination of Carbohydrate Content

#### 2.4.1. Quantitative Determination of Carbohydrate Content

Total carbohydrate content contained in the test portion of *A. platensis* was calculated using the formula:

Carbohydrate content = 100 − (humidity + mineral matter + fat + proteins) [21].

#### 2.4.2. Qualitative Determination of Carbohydrate Content

Five grams of *A. platensis* sample was extracted with 50 mL of demineralized water for 2 hours. Afterward, the carbohydrate concentration of the filtrate obtained was dosed using HPLC (column: silica grafted with NH_2_: 25^*∗*^4, 6^*∗*^5 cm^*∗*^mm^*∗*^*µ*m; mobile phase: acetonitrile/water (80/20, respectively); flow rate: 1 mL/min) [21].

### 2.5. Fat Determination

#### 2.5.1. Quantitative Determination

Five grams of *A. platensis* sample was mixed with 31.5 mL of hydrochloric acid supplemented with 125 mL of distilled water for 2 hours (12 N). After filtration, the residues obtained were placed in an oven set at 105°C overnight. Next, the residues obtained were extracted again with 300 mL petroleum ether using a Soxhlet for 4 hours [21].

After removing the solvent under reduced pressure, the measures were performed using the following formula:

(3) $$\begin{array}{c} \text{Fat content }\left(\%\right)=\frac{T2-T1}{\text{PE}}\times 100. \end{array}$$

Here, *T1*: the weight of empty flasks, *T*2: the weight of flasks containing fat, and PE: test portion.

#### 2.5.2. Analysis of Fatty Acid Composition

The fatty substance was esterified with methanol. Next, the fatty acid methyl esters were separated through a polar column using gas chromatography (GC) (Annex 5). Briefly, 2 mL of isooctane and 0.1 mL of methyl KOH (2N) were added to 0.5 g of the extracted fat to prepare methyl esters. Afterward, the mixture was stirred for one minute before adding 2 mL of NaCl (40 grams/100 mL). Next, one gram of sodium bisulfate was added to the recovered supernatant before proceeding with the gravimetric analysis [21].

### 2.6. Determination of Minerals and Heavy Metals

#### 2.6.1. Dosage of Cu, Fe, Mn, Ca, Mg, K, Na, Pb, Cr, Cd

Concentrations of calcium (Ca) and magnesium (Mg) were determined by adding 2 mL of lanthanum chloride La_2_O_3_ (50g/L) to the mother solution. Potassium (K) determination was conducted by adding 2 mL of cesium chloride. Next, the mineral content was measured using atomic absorption spectrophotometry with flame (Varian SpectrAA 220FS Spectrometer FLAME AA with Varian SIPS-10 Sample Introduction Pump System with Varian SPS-5 Sample Preparation System) [21].

#### 2.6.2. Dosage of P

One milliliter of mother solution was mixed with 10 mL of the monovanado-molybdic reagent. The analysis was conducted using a UV spectrophotometer at 430 nm [23]. The mineral concentration was calculated according to the following formula:

(4) $$\begin{array}{c} \text{Percentage of minerals}=\left(L-B\right)\times 10^{-3}\times \frac{\text{VR}}{1000}\;\times \frac{100}{\text{PE}}\times \text{FD}. \end{array}$$

Here, *L*: reading, *B*: blank, VR: recovery volume, PE: test portion, and FD: dilution factor (g).

### 2.7. Determination of Vitamins

Vitamins B_2_ (riboflavin) and B3 (nicotinamide) contained in *A. platensis* were determined by using HPLC (NM 08.1.264 (2009). Briefly, 2 g of *A. platensis* sample was added to 40 mL of sulfuric acid (0.1 mol/L) before being stirred for 15 min. Afterward, the mixture was completed with sulfuric acid to reach 100 mL as a final volume. The concentration of vitamins was measured with HPLC (mobile phase: mixture of 970 mL of n-octane sulfonic acid 7 mmol/L (pH = 3), 30 mL of acetonitrile; stationary phase: Hypersil HyPURITY C18 5 *µ*m 250^*∗*^4.6 mm; injection volume: 50 *µ*L; flow rate: 1 mL/min; fluorimetric detection: 375nm–525 nm; UV detection: 261 nm; gradient in min: 0-9-9.1-22-22.1 and 35).

The concentration of vitamins B_2_ and B_3_ was calculated using the following formula:

(5) $$\begin{array}{c} \text{Percentage of vitamins}=\frac{\text{AEch}}{\text{ASt}\times \text{FD}\times \text{CSt}}. \end{array}$$

Here, AEch: peak area of the sample, ASt: peak area of the standard solution, FD: dilution factor = (final volume/test sample), and CSt: concentration of vitamin standard solution (0.2 for B2 and 5 for B3) [21].

### 2.8. Energy Value Determination

The evaluation of *A.* platensis energy value was based on the calorific value of different components (proteins, lipids, and carbohydrates) [24].

The energy value was calculated according to the following formula:

(6) $$\begin{array}{c} \text{Energy Value }\left(\frac{K\text{cal}}{100\;g}\right)=\left(4\text{ Carbohydrates }+\;9\text{ Fat }+\;4\text{ Proteins}\right)\times 100. \end{array}$$

### 2.9. Humidity Determination

Two grams of *A. platensis* were placed in previously weighed glass capsules before being introduced into an oven set at 105°C for 4 h. Next, the humidity percentage was calculated according to the following formula:

(7) $$\begin{array}{c} \text{Humidity percentage}=\frac{\left(T2-T3\right)}{\left(T2-T1\right)}\times 100. \end{array}$$

Here, *T*1: the weight of the empty capsule, *T*2: the weight of capsule containing the fresh sample, and *T*3: the weight of capsule containing the dry sample [25].

### 2.10. Ash Content Determination

Three grams of *A. platensis* sample was introduced into a capsule before being placed in a muffle oven set at 550°C for six hours [26]. After cooling for fifteen minutes, the remaining mineral matter was weighed. The ash content was calculated according to the following formula:

(8) $$\begin{array}{c} \text{Ash content}\left(\%\right)=\frac{\left(T3-T1\right)}{\text{PE}}\times 100. \end{array}$$

Here, *T*1: the weight of empty capsule, *T*2: the weight of capsule containing the fresh sample, *T*3: the weight of capsule containing the dry sample, and PE: test sample = *T*2 − *T*1.

### 2.11. Determination of Pigments

The content determination of carotenoids, chlorophyll a, and chlorophyll b was done according to the earlier reported data [27]. Briefly, 0.5 g of *A. platensis* sample was extracted with 10 mL of acetone under ultrasound (130 W, 20 KHz) for 15 min. After filtration, the mixture was centrifuged at 3000 rpm for 10 min. The pigment content (carotenoids, chlorophyll a, and chlorophyll b) was determined according to the following formula:

(9) $$\begin{array}{c} \text{Chl }a=13.36\times A664-5.19,\\ \text{Chl }b=27.43\times \text{A}648-\;8.12\text{,}\\ \text{content of carotenoids}=\frac{\left(1000\times A470-1.63\times \text{chl }a-104.96\times \text{chl}b\right)}{\text{Chl }a:\text{Chlorophyll }a,\text{Chl }b:\text{ Chlorophyll }b}. \end{array}$$

### 2.12. Determination of Tannins

The content of tannins was determined according to the previously reported method [28].

### 2.13. Statistical Analysis

Data were expressed as means of triplicate assays ± SD (standard deviation). The significant differences were investigated using the *t*-test. The Mann–Whitney test was used as a post doc test to perform the comparison. Statistically, a significant difference was considered at *P* < 0.05.

## 3. Results and Discussion

### 3.1. Protein Content Determination

The value of protein content in *A. platensis* was 58.9 ± 0.07%. Besides, this species was quantitatively rich in essential amino acids as reported in our findings (Table 1). The characterization of *A. platensis* total amino acid content with high-performance liquid chromatography showed the presence of threonine and phenylalanine as major constituents of essential amino acids. Methionine was the most sulfur amino acid detected in the studied species. *A. platensis* also found to be rich in nonessential amino acids like glutamic acid (Table 1).

In the current research work, we investigated *A. platensis* as one of the most recommended foods to prevent diseases. The findings of chemical composition showed that *A. platensis* collected from the South Atlantic Coast of Morocco has a significant amount of proteins (58.9 ± 0.07%). This species contains protein twice as much as soybeans and thrice as much as meat [29, 30]. A promising rate of essential amino acids was found in the investigated species including glutamic and threonine acids (5042.29 ± 374.19 mg/kg and 131.39 ± 1.96 mg/kg, respectively), which quantitatively exceeded those reported in species from different cultivars [30]. Our results showed that the methionine content in local *A. platensis* was 43.71 ± 0.68 mg/kg. Therefore, we can confirm that *A. platensis* collected from the Moroccan Atlantic coast at Laayoune belongs to the rare cyanobacteria that contain a high amount of methionine. These results agree with those reported elsewhere since it was stated that *Arthrospira* constitutes a promising source of protein not only because of its high rate in the dry biomass but also due to the composition of amino acids and high digestibility. It is worth reporting that *Arthrospira* biomass possesses all 8 exogenic and 12 endogenic amino acids [31].

### 3.2. Determination of Carbohydrate Content

High-performance liquid chromatography analysis showed that the value of carbohydrate content in dry *A. platensis* was 14.67 ± 0.001%. The characterization of *A. platensis* total carbohydrate content showed the presence of a significant amount of glucose (3.17 ± 1.01 g/L), unlike fructose, sucrose, and maltose, which were detected in very little quantity.

The present results showed that *A. platensis* has a low content of carbohydrates (14.67 ± 0.001%) with a very little amount of simple carbohydrates (glucose and fructose). These findings were in agreement with those reported in earlier works [32]. *Arthrospira* was described by the presence of two specific polysaccharides including sodium spirulan and calcium spirulan, which are involved in antiviral, anticoagulant, and immunostimulatory activities of *A. platensis* as reported elsewhere [31, 33].

The total lipid content of *A. platensis* was determined at 5.8 ± 0.21% using gravimetric analysis. The characterization of *A. platensis* total lipid content revealed the presence of monounsaturated and saturated fatty acids with 53 ± 0.003% and 45.54 ± 0.15%, respectively. The total lipid content of the presently studied species was majorly constituted of palmitoleic acid (45.52 ± 0.01%), palmitic acid (37.06 ± 0.502%), and oleic acid (7 ± 0.003%) (Table 2).

### 3.3. Fat Determination

Regarding the total lipid content in the local *A. platensis,* the results obtained showed that this species was majorly constituted of saturated and unsaturated fatty acids with a total percentage of 5.8 ± 0.25%. *A. platensis* possessed both monounsaturated and saturated fatty acids with values of 53 ± 0.003% and 45.54 ± 0.15% respectively. Our findings are in accordance with those reported in earlier works, which showed that lipid in *Arthrospira* varies from 1.5 to 12% of dry mass [34].

### 3.4. Determination of Minerals and Heavy Metals

The mineral composition analysis with flame atomic absorption spectroscopy revealed that *A. platensis* collected from the South Atlantic Coast of Morocco have important mineral elements (Table 3) and very few or no heavy metals (Table 4). The values of lead and cadmium content in *A. platensis* were lower than the largest tolerated values in the food according to the World Health Organization (WHO). Statically, there was a significant difference between the content of heavy metals detected in *A. platensis* and WHO threshold values (*P* < 0.05). Moreover, *A. platensis* was free of cadmium as shown in Table 4.

*Arthrospira* largely meets the nutritional requirements for the body since it is rich in essential mineral elements. The present findings showed that this species has important mineral elements (Ca, Mg, K, Na, P) (Table 4). Therefore, we can confirm that these results were in accordance with those reported in earlier works [29]. Our species was screened for potential heavy metals whose results showed very few or no heavy metals (Fe, Zn, Mn, Cu) (Table 5). According to the results obtained in the present study and those previously reported on the phytochemical screening of *A. platensis*, we can confirm that this species is rich in minerals, and therefore, *A. platensis* can be considered a good choice for nutritional supplement product since the minerals discussed in this study play an important role in the function of the body. More specifically, potassium (K) helps prevent hypertension and improve bone health, whereas phosphorus (P) is required for skeletal mineralization [35]. Furthermore, magnesium (Mg) is a cofactor for a variety of metabolic activities and is essential for bone mineralization and muscle relaxation [36], and iron (Fe) prevents anemia by generating hemoglobin and myoglobin. It is also involved in the production of enzymes and other iron-containing enzymes [37].

Lead and chromium were found to be present in the local species with values of 70 ± 4.5 PPB and 5 ± 0.5 PPB, respectively; however, no trace was detected for cadmium. These values are lower than those of the World Health Organization threshold (mercury, 5 *µ*g/kg/week; lead, 25 *µ*g/kg/week; cadmium 7 *µ*g/kg/week).

### 3.5. Determination of Vitamins

The results obtained showed that *A. platensis* possesses vitamins B_2_ and B_3_ with values of 1.31 ± 0.19 and 30.8 ± 0.001 mg/kg, respectively, according to the high-performance liquid chromatography analysis (Table 5).

Since it contains significant levels of fat-soluble vitamins (vitamins A, D, E, and K) and water-soluble vitamins (vitamin B_2_: 1.31 ± 0.19 mg/kg; vitamin B_3_: 30.8 ± 0.001 mg/kg), *A. platensis* could cover the requirements of vitamins, which the body is unable to synthesize. Thus, these results were in agreement with those reported in earlier literature [38].

### 3.6. Energy Value Determination

The findings of energetic values assessed in the current research work showed that *A *. *platensis* from the local cultivar possesses a high energetic value (346.48 ± 0.21 kcal/100 g). The remarkable value of total lipid content (5.8 ± 0.25%) found in A*. platensis* could be responsible for its high energetic value (348.6 ± 0.21 kcal/100 g). In this sense, arachidonic as derived acid from palmitic acid plays a key role in the synthesis of prostaglandins and leukotrienes [39]. Moreover, the findings of chemical analysis showed that the studied species was found to be higher in polyunsaturated fatty acids including omega-3 and 6 that are involved in the prevention of cholesterol accumulation in the body [38, 39].

### 3.7. Moisture Determination

The findings obtained in the current research showed that the value of moisture content determined in the studied organism was 11.6% ± 0.17. Moisture content is defined as a quantity of water that exists in the biomass. Moisture plays a key role in food storage due to its either direct or indirect effect on microorganism development. In the present work, the moisture content defined in *A. platensis* was 11.6% ± 0.17. Therefore, these results were partially in agreement with those stated in earlier works, which showed that the moisture content in *A.* platensis was 12.5% corresponding to 56% relative humidity [40].

### 3.8. Ash Content Determination

The results reported in the present study showed that the ash content determined in *A. platensis* was estimated at 9.1% ± 0.21. The ash is a measure of mineral content in biomass. In food, ash content is an important part of food quality analysis. Herein, *A. platensis* was also investigated in terms of ash content. As reported in the current research, the ash content was estimated at 9.1% ± 0.21. This finding was supported by the earlier found data, which reported that *Arthrospira* grown in Zarrouk's medium acquired the highest percentage of ash [41].

### 3.9. Determination of Pigments

The analysis of pigment content in *A. platensis* showed the presence of chlorophyll *a*, chlorophyll B, and carotenoid with values of 26.066 ± 3.08 mg/g, 37.506 ± 3.38 mg/g, and 9.52 ± 0.22 mg/g, respectively. Regarding the pigment production, the analyzed sample evidenced the presence of chlorophyll b, chlorophyll a, and carotenoids with values 37.506 ± 3.38 mg/g, 26.066 ± 3.08 mg/g, and 9.52 ± 0.22 mg/g, respectively. Thus, these findings were in accordance with those reported in earlier works, which showed that the values of chlorophyll and carotenoid content in the genus *Arthrospira* were 26 mg/g and 3 mg/g DM, respectively. *Arthrospira* cells possessing carotenoids in different forms including *α*-carotene, *β*-carotene, cryptoxanthin, zeaxanthin, xanthophylls, echinenone, and lutein as reported elsewhere [32]. Therefore, we can confirm that this species can be a promising source of pigments like chlorophylls, carotenoids, and phycocyanins as reported in the earlier literature [31].

### 3.10. Determination of Tannins

Qualitative analysis of *A. platensis* extracts revealed a low tannin content. Gallic tannins were also present in little amount. Tannins are belonging to the secondary metabolites synthesized by plants and microorganisms to accomplish ecological functions. Our results showed that our organism has no important amount of tannins. Hence, these results were in contrast with the previously reported literature, which revealed the presence of promising tannin content in the genus *Arthrospira* [6].

### 3.11. Comparison of *A. platensis* Indigenous to the Moroccan Atlantic Coast at Laayoune with the Same Species from Different Collection Areas in Terms of Physicochemical Characteristics

**Figure 3:**
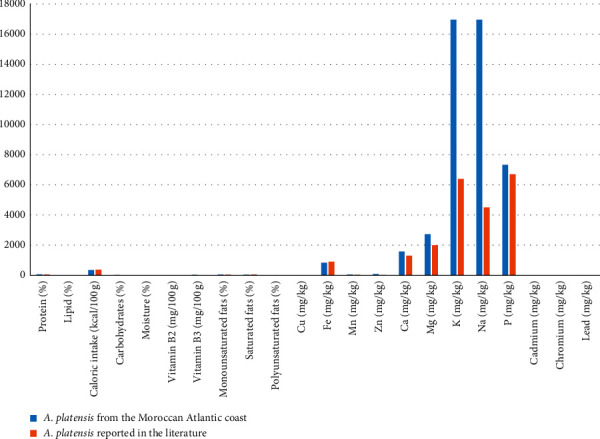
Physicochemical characteristics of *A. platensis* indigenous to the Moroccan Atlantic coast at Laayoune in comparison with those of the same species reported in the literature.

Species of *A. platensis* indigenous to the Moroccan Atlantic coast at Laayoune possess unique features in terms of physicochemical contents when compared with the same species collected from different collection areas as reported in earlier works [25]. The studied species in the present work was found to be higher in the following parameters: proteins, carbohydrates, monounsaturated fats, moisture, vitamin B_2_, vitamin B_3_, ash, Mn, Zn, Ca, Mg, K, Na, and P when compared with the same species indigenous to other areas. Our species was also found to be lower in heavy metals (lead, chromium, and cadmium) and saturated fats as nonrequired parameters in foods (Figure 3; Table 6).

Malnutrition is a public health problem throughout the world over the past decades. Several people worldwide have suffered malnutrition and food-related chronic diseases. In Africa, more than 30% of the deaths of less than five-year-old children result directly or indirectly from malnutrition, which is coupled with deficiencies in vitamins and minerals. It is thus fitting that people across the world have looked for natural products to improve health or to remedy deficiencies. Around fifty microalgae are currently consumed worldwide. The most common in the trade are sea lettuce, dulse, sea beans, nori, wakame as well as spirulina, and chlorella. [2]. The consumption of spirulina as a portion of food could back to many years ago. The nutritional value of spirulina can be due to its chemical composition, which is constituted of fibers, minerals, and proteins in large part, not that only but also the presence of secondary metabolites (vitamins, tannins), which are known to possess antioxidant and antibacterial effects. The chemical composition of spirulina exhibits other important benefits such as cosmetic, pharmacological, and therapeutic values [6].

Our results are in accordance with those reported by Jourdan, who showed that *A. platensis* possessing about 50 to 70% protein, 15 to 25% carbohydrates, and 11% for lipids, vitamins, minerals, as well as chlorophyll [44]. In this sense, it was reported that *A. platensis* is a potential source of several water-soluble vitamins (B2, B3, B5, and B9), which act as coenzymes for mitochondrial enzymes and play important roles in cell metabolism and energy production according to prior research [45].

*A. platensis* possesses an interesting protein family that is recognized by its activities such as antioxidant, anticoagulant, antihypertensive, immunomodulatory, and antimicrobial [29]. Species of *A. platensis* indigenous to the Moroccan Atlantic coast at Laayoune have unique features in terms of physicochemical contents when compared with the same species investigated elsewhere. The obtained results showed that our studied species were higher in proteins, polyunsaturated and monounsaturated fats, minerals, vitamins (B_2_, B_3_), ash, and pigment contents when compared with species studied by Bensehaila, (2015). Moreover, our species was lower in heavy metals and saturated fats when compared with those studied by Bensehaila, (2015), and Falquet, (2012). Therefore, we could confirm that *A. platensis* indigenous to the Moroccan Atlantic coast at Laayoune is a promising source of food supplement due to their high values concerning proteins, unsaturated fats, carbohydrates, minerals, vitamins, and pigments and their low values regarding heavy metals and saturated fats. *A. platensis* is reported to be of high nutritional value and pharmacological and biological potentials so that they can be used for medicinal purposes for humans or animals [46]. In addition to their nutritional and pharmacological properties, *A. platensis* has a biofuel production potential, and therefore, it can significantly contribute to economic performance [47].

*A. platensis* cannot be free of toxins. In this sense, the rate of trace elements (B, Ba, Li, Ni, Sr, V) alongside toxic metals (Al, Cd, Pb) was detected in *A. platensis* samples. The highest element concentration was detected in the powder format, except for Li. When element levels in *A. platensis* exceed the tolerable weekly intake (TWI), the consumer would place his health at risk. The consumption of spirulina contributes largely to the Al intake by a value higher than TWI determined at 1 mg/kg bw/w (body weight/week), followed by Cd exceeding its TWI set at 2.5 *μ*g/kg bw/w was reported in previous works [48]. Pb intake with a value higher than the TWI level can be associated with nephrotoxicity and cardiovascular effects. However, this literature suggests that spirulina consumption does not place the consumer at risk as far as exposure to toxic metals (Al, Cd, Pb) is regarded as a concern. However, the presence of trace elements and toxic metals in spirulina destined for food purposes should be monitored to ensure its quality and safety. In contrast, lead and chromium were found in our species with values of 70 ± 4.5 PPB and 5 ± 0.5 PPB, respectively; however, no trace was detected for cadmium. These values are lower than those of the World Health Organization threshold (mercury, 5 *µ*g/kg/week; lead, 25 *µ*g/kg/week; cadmium 7 *µ*g/kg/week). Therefore, *A. platensis* indigenous to the Moroccan Atlantic coast at Laayoune (Foum El Oued lagoon) can be considered safe for being ingested.

## 4. Conclusion

The present research work aims to assess the nutritional value of *A. platensis* collected from the Moroccan Atlantic coast at Laayoune. The obtained results showed that *A. platensis* indigenous to the Moroccan Atlantic coast at Laayoune was found to be very rich in proteins, carbohydrates, vitamins, minerals, ash, and pigments and lower in heavy metals and saturated fats when compared with species investigated in the literature. Therefore, we could confirm that *A. platensis* indigenous of the Moroccan Atlantic coast at Laayoune can be a very promising source of dietary supplements. Overall, *A. platensis* should be optimized further, and processing strategies based on the optimization approaches can be developed.

## Figures and Tables

**Table 1:** Amino acids content in *A. platensis*.

Amino acid	Content in mg/kg
Methionine	43.71 ± 0.68
Threonine	131.39 ± 1.96
Phenylalanine	44.97 ± 2.64
Valine	63.05 ± 4.73
Arginine	34.82 ± 1.15
Glutamic acid	5042.29 ± 374.19

**Table 2:** Total lipid content in *A. platensis*.

Compound name	Percentage (%)
Lauric acid	0.86 ± 0.01
Myristic acid	1.90 ± 0.41
Palmitic acid	37.06 ± 0.502
Palmitoleic acid	46.52 ± 0.15
Margaric acid (17:0)	4.6 ± 0.03
Margaric acid (17:1)	0.25 ± 0.001
Stearic acid	0.9 ± 0.01
Pentadecylic acid	0.23 ± 0.0005
Vaccenic acid	7 ± 0.003
Linoleic acid	1.20 ± 0.001
*γ*-Linolenic acid	0.26 ± 0.001
Arachidic acid	0.05 ± 0.01
Gondoic acid	0.23 ± 0.0001

**Table 3:** Mineral elements contained in *S. platensis*.

Minerals	Wavelength	Content in mg/kg
Cu	324.8	6.95 ± 0.07
Fe	248.3	836.7 ± 131.8
Mn	279.5	47.28 ± 1.17
Zn	213.9	89.7 ± 54.7
Ca	423	1579.22 ± 68.7
Mg	202	2729.71 ± 46.25
K	767.2	16954.85 ± 305.8
Na	589.6	
P	430	16954.85 ± 29.2

**Table 4:** Heavy metals detected in *A. platensis*.

Heavy metals	Content in PPB	Max. Level in PPB (WHO)
Lead	70 ± 4.5	658
Cadmium	0	472
Chromium	5 ± 0.5	30

**Table 5:** Vitamins detected in *A. platensis*.

Sample	Percentage (%)
Standard Vit B_3_	100.000
Standard Vit B_2_	53.687
Sample Vit B_2_	18.0055
Sample Vit B_3_	55.836

**Table 6:** Comparison of *A. platensis* indigenous to the Moroccan Atlantic coast with the same species from different collection areas in terms of physicochemical characteristics.

Parameter	*A. platensis* indigenous to the Moroccan Atlantic coast	*A. platensis* reported in the literature	Publications used
Protein (%)	58.9 ± 0.07^a^	52.86 ± 2.92^a^	[25]
Lipid (%)	5.8 ± 0.25^*a*^	7.28 ± 0.021^*a*^	[25]
Calorie intake (kcal/100 g)	346.48^*a*^	369.28^*a*^	[25]
Carbohydrates (%)	14.67 ± 0.001^*a*^	13.6^*a*^	[25]
Moisture (%)	11.6% ± 0.17^*a*^	5.42 ± 0.031^*b*^	[25]
Vitamin B_2_ (mg/100 g)	1.31 ± 0.19^*a*^	0.009^*b*^	[42]
Vitamin B_3_ (mg/100 g)	30.8 ± 0.001^*a*^	0.053^*b*^	[42]
Monounsaturated fats (%)	53 ± 0.003^*a*^	40.1^*b*^	[42]
Saturated fats (%)	45.54 ± 0.15^*a*^	55.72^*b*^	[43]
Polyunsaturated fats (%)	1.46 ± 0.01	—	[42]
Cu (mg/kg)	6.95 ± 0.07^*a*^	8^*a*^	[42]
Fe (mg/kg)	836.7 ± 131.8^*a*^	900^*a*^	[42]
Mn (mg/kg)	47.28 ± 1.17^*a*^	25^*b*^	[42]
Zn (mg/kg)	89.7 ± 54.7^*a*^	21^*b*^	[42]
Ca (mg/kg)	1579.22 ± 68.7^*a*^	1300^*b*^	[42]
Mg (mg/kg)	2729.71 ± 46.25^*a*^	2000^*b*^	[42]
K (mg/kg)	16954.85 ± 305.8^*a*^	6400^*b*^	[42]
Na (mg/kg)	16954.85 ± 29.2^*a*^	4500^*b*^	[42]
P (mg/kg)	7335.35 ± 123.6^*a*^	6700^*b*^	[42]
Cadmium (mg/kg)	0.005 ± 0.0005^*a*^	14.2^*b*^	[42]
Chromium (mg/kg)	0	≤1	[42]
Lead (mg/kg)	0.07 ± 0.0045^*a*^	5 ± 00.5^*b*^	[42]

The results were given as average ± standard deviation. The reported values with the same letter in the same line did not differ significantly at *P* < 0.05.

## Data Availability

Data used to support the findings are included within the article.
